# Mental Health Provision in UK Secondary Schools

**DOI:** 10.3390/ijerph182212222

**Published:** 2021-11-21

**Authors:** Megan Garside, Barry Wright, Roshanak Nekooi, Victoria Allgar

**Affiliations:** 1 COMIC Research, Leeds and York Partnership NHS Foundation Trust, York YO10 5NP, UK; roshanak.nekooi@nhs.net; 2 Department of Health Sciences, University of York, York YO10 5NP, UK; barry.wright1@nhs.net; 3 Faculty of Health, Peninsula Medical School, University of Plymouth, Plymouth PL4 8AA, UK; victoria.allgar@plymouth.ac.uk

**Keywords:** adolescent, children, education, mental health, schools, wellbeing, teachers

## Abstract

Research reports high levels of mental health problems faced by young people in the UK. Schools provide a range of mental health support services, although these are often not robustly evaluated. This paper aims to explore the mental health provision of secondary schools across two large regions in the North of England and provide comparisons to the mental health questionnaire scores of their pupils. Results are part of a wider study providing an overview of the mental health of secondary school pupils. Measures include the Strengths and Difficulties Questionnaire, distributed to year 8, 9, and 11 pupils attending secondary schools and a bespoke mental health service provision questionnaire for school staff at the same schools. A total of 6328 pupil questionnaires and 36 staff questionnaires were returned from 21 schools. Results showed a non-significant correlation between provision and young people’s mental health scores and highlight a range of factors to take into consideration. There is a need to improve the evaluation and recording of school-based mental health provision. Mental health difficulties in young people are prevalent in schools. Future research is needed to elucidate which types of services are most helpful in preventing, supporting, and signposting those with mental health problems.

## 1. Introduction

There has been a reported gradual rise in mental health problems in children and young people since 2004, as found through a major survey commissioned by NHS Digital [1]. This survey report outlined that one in seven (14.4%) 11–16-year-olds surveyed in 2017 were diagnosed with a mental health disorder using a multi-informant measure, compared to one in ten (10%) in 2004 [2]. Emotional disorders were the most common (9%), followed by behavioural difficulties. Results from a follow-up wave to this survey have shown that these mental health problems are continuing to rise, with one in six (16%) children aged 5 to 16 years old identified as having a probable mental disorder [3]. This increase has been noted by other researchers [4,5], although in contrast, a study conducted before the COVID-19 pandemic that looked in detail at trends across selected mental health outcomes found no increase in emotional disorders in young adolescents in England [6].

A recent independent Child and Adolescent Mental Health Service (CAMHS) review has highlighted that there has been a 26% increase in referrals over the past 5 years. The average waiting time for treatment had a median in 2017–2018 of 34 days to assess and 60 days to treat [7]. Vulnerable children require access to timely, person centred, high-quality care in order to improve their mental health and to prevent further deterioration [8,9,10].

Teachers provide valuable information about the mental health of young people [11] and about two thirds of young people with a psychiatric disorder report contact with a teacher about their mental health [12]. Over recent years, there has been an emphasis on the role of schools in providing evidence-based early intervention support [13,14] with targeted mental health support in schools proving helpful for some sub-groups [15]. The 2017 Government Green Paper outlined the need for a boost in support for the role schools and colleges play in child and adolescent mental health [8]. Research has shown school-based services can provide early intervention for mental health support [16,17,18]. Schools can support children through education about mental health, supporting development of resilience and providing strategies for managing mental health. This can help by providing early support and intervention before reaching the level of need of specialist services.

One way in which schools are encouraged to do this is by adopting a whole school approach (WSA), whereby a culture is established in which universal and targeted approaches can thrive [19]. A whole school approach requires partnership between leaders at the school, teachers, parents and the wider community [20]. Reviews support the use of the whole school approach for positive impacts on pupil mental health. This has been suggested to be a result of the change in culture within the school and involvement of staff, parents, and community services having a wide impact [21]. A whole school approach has been defined as including universal and targeted approaches to supporting mental health by embedding promotion, prevention, and early intervention activities for mental health [19]. Universal approaches include providing training for staff to identify mental health problems and developing partnerships with parents, families, and community services. Targeted approaches include externally provided therapeutic support such as counselling, voluntary agency provision, or mental health worker support and a range of internal provision such as one-to-one sessions with an internally trained staff member and targeted individual or small group interventions (e.g., anger management) [22,23]. A recent systematic review investigated whole school approach interventions [24]. This study looked at the presence or absence of key characteristics including teacher training, available programs or interventions to enhance social and emotional skills, strategies addressing the whole school ethos and environment (e.g., strong bullying policies and peer mentor schemes), parent and community involvement, and targeted support for those at risk of developing emotional or behavioural problems. Results showed a significant but small improvement in outcomes including social and emotional adjustment. However, the evidence was limited, with only two of the forty-five included studies based in the UK and not all studies reported social and emotional outcomes.

Schools differ in terms of the mental health support they provide, partly related to degree of identified need, financial challenges, capacity, and prioritisation decisions [25]. This can influence pupil and staff mental health outcomes, although up to date evidence evaluating outcomes for these approaches is limited. There is a limited evidence base for UK school-based services and a need for more clarity on what is delivered and how [26].

This paper aims to explore school-based mental health provision (reported by staff and based on comparisons to elements of a whole school approach) and compare this to the mental health scores from their year 8, 9, and 11 secondary school-aged pupils. It was expected that there would be a relationship between school provision and pupil mental health scores. This is based on the existing literature which suggest school-based services can provide early mental health support, particularly when they incorporate a whole school approach.

## 2. Materials and Methods

### 2.1. Design

This large-scale evaluation is a cross-sectional design presenting questionnaire data collected from young people attending secondary schools across the North of England during the 2017/2018 academic school year. Participating school staff completed questionnaires regarding the mental health services provided by their school. Results presented here are part of a wider study, reported elsewhere [27].

Ethical approval was obtained from the University of York Health Sciences Research Ethics Committee (HSRGC/2017/246/A).

### 2.2. Participants

A total of 21 schools participated and questionnaires were returned from 6328 pupils in years 8, 9, and 11 at these schools. The age range was selected as 11–16 which is the age group included in most large studies of mental health in the UK [1] and covers the age when most mental health problems develop. Year 11 captures the upper end of this range and year 8 is first year post transition from primary school. We included year 8 as the lower end of this range as those pupils in year 7 will have only recently transitioned from primary school and will not have had a full experience of the mental health services offered at their school.

Senior staff members with responsibility for mental health support and/or pastoral care at each school completed a questionnaire detailing mental health provision. We provided 3 questionnaires per school so that multiple members of staff could complete one. In total, 36 questionnaires were completed, with all 21 schools returning at least one. Where multiple staff completed questionnaires from the same school, we cross-checked responses and if any discrepancies were identified, we contacted the school via telephone for clarification. We compared these scores to the year 8, 9, and 11 pupils attending these 21 schools in the 2017/2018 academic year (*n* = 6328).

### 2.3. Measures

The wider study collected a range of mental health outcome measures (including EQ-5D-Y, social media use, health service resource use) reported elsewhere [22]. Here we report on the service provision questionnaire completed by school staff and the Strengths and Difficulties Questionnaire (SDQ) completed by pupils.

#### 2.3.1. Strengths and Difficulties Questionnaire (SDQ)

The self-report SDQ (11–17 years old) with impact supplement [28] was used to record pupil mental health information. The SDQ presents 25 statements, to which the young person selects the most appropriate response from ‘Not True’, ‘Somewhat True’, or ‘Certainly True’ based on how they feel it represents themselves over the past 6 months. These 25 questions cover 5 subscales. There are four difficulty subscales: emotional problems, conduct problems, hyperactivity, peer problems, and one reverse-scored strength scale, prosocial behaviour. The four difficulty subscales can be combined to give a total difficulties score (TD) [29]. Scores on each of the subscales can be categorised as ‘Close to Average’, ‘Slightly Raised’, ‘High’, or ‘Very High’, with thresholds based on a UK-based population survey [30]. The SDQ is commonly used in mental health services and has a range of research supporting its consistency and validity [31].

#### 2.3.2. Mental Health Provision Questionnaire

Senior staff completed a bespoke questionnaire detailing the mental health provision at their school during the current academic year. This bespoke questionnaire was not validated and was developed with a study management group (with experts within the field including academics, teachers, and local authority staff). Follow-up phone calls were used to collect missing information.

Six common support areas were identified from previous literature [19,22,23,24] and given a score based on how they were implemented. These items map on to those areas identified as part of the whole school approach in the literature. A study management group panel (including input and advice from academics, teachers, and local authority staff) prospectively decided the questionnaire and scoring system, described in Table 1. The total overall mental health provision score (TOMPS) for each school is a ‘service provision score’ (possible range 0–12) derived from this questionnaire.

The questionnaire also contained questions about links with their local CAMHS and free text response space to identify if there was any other support, services, or training staff felt was needed.

### 2.4. Procedure

All secondary schools across two large regions in the North of England were eligible to participate. All eligible schools were contacted via email and phone call to invite them to participate. Early in the spring term, head teachers of eligible secondary schools were contacted. If the schools agreed to participate, eligible pupils (attending years 8, 9, and 11) were given participant information sheets about the study. Opt-out consent was used. Schools notified parents electronically by text, email, or newsletter. Pupils were able to opt-out in a number of ways: by returning a form or other contact from their parents/carers or by their own choice on the day.

Research assistants printed and delivered the correct number of required questionnaires to schools. They also provided instructions to support the school staff with organising the questionnaire completion. The schools then distributed the questionnaires to classes, where pupils completed them within school time, during a PSHE lesson, or similar. The research assistant then collected the questionnaires from each school.

Questionnaires were completed in the spring term, in 2018. We collected data from year 8, 9, and 11 pupils across 21 schools. Schools were kept anonymous and all pupil questionnaires were assigned a unique alphanumeric ID code to ensure confidentiality.

Raw scores were entered onto a secure database. Five percent of the whole data set was randomly selected and cross-checked by a second research assistant. No systematic errors were found. For any school that had more than one error, 10% of this school was checked. If any further errors were found in this second check, all questionnaires were checked and re-entered.

### 2.5. Analysis

All analyses were undertaken on SPSS [32]. Descriptive data, presented as mean (and standard deviation) or n (%), is provided on the staff responses to the questionnaires regarding school provision and for the self-reported pupil Strengths and Difficulties Questionnaire (SDQ). A Spearman’s Rank correlation was used to examine associations between school service provision score and the SDQ scores from their year 8, 9, and 11 pupils.

## 3. Results

### 3.1. Overview

Table 2 shows the demographic information for the total number of pupils in each year group completing the Strengths and Difficulties Questionnaire (SDQ). Where pupils did not fill in details on the form, this was marked as ‘not completed’.

Approximate number of pupils ranged in each school from 100 to over 2000. Community schools, free schools, foundation schools, academies, and voluntary-controlled and voluntary-aided schools were included. Five schools were religious; sixteen were non-religious. The percentage of pupils eligible for pupil premium in each school ranged from under 10% to approximately 40% [33].

From the 21 schools, there was a total cohort of approximately 9896 eligible pupils, meaning a 64% response rate was achieved with the 6328 returned questionnaires.

There were approximately 84 eligible schools. One school withdrew due to capacity issues and 12 decided not to participate (due to capacity, staff changes, and competing demands from another study). All other schools were contacted several times via telephone and email but did not respond within the recruitment timeframe. Our final sample was diverse in terms of socio-economic and geographical factors.

### 3.2. Strengths and Difficulties Questionnaire (SDQ)

The overall scores on the SDQ subscales are shown in Table 3, as well as the proportion of those scoring within the ‘Very High’ range. Further detail regarding pupil self-reported measures can be found reported elsewhere [27]. Results showed almost 1 in 10 pupils were scoring in the ‘Very High’ range on the total difficulties subscale (9%), with an overall mean score of 11.3. On the subscales, approximately 1 in 7 pupils were scoring in the ‘Very High’ range for the emotion subscale (14%).

### 3.3. Mental Health Service Provision Questionnaire

The responses to the staff questionnaire are summarised in Table 4. As described above, the common support items were selected based on current literature and included elements which map on to a whole school approach.

Over half of schools reported long waiting lists for access to CAMHS (57%) and a third specifically highlighted a need for increased support from and improved links with CAMHS, although recognised capacity and funding as barriers.

Over half of schools (57%) identified the need for a dedicated 1:1 mental health worker in their school. Over a quarter (29%) of schools identified a need for a school counsellor, including schools that already had this service, highlighting a need for more consistent availability from them.

### 3.4. Number of Services

The total overall mental health provision score (TOMPS) for services provided at each school based on their responses to the staff questionnaire were compared to mean SDQ total difficulties scores completed by year 8, 9, and 11 pupils attending these schools in 2018 (*n* = 6328). A Spearman’s correlation found the correlation between mean total difficulties and TOMPS as rs −0.280, *p* = 0.218, suggesting a potential weak relationship between provision and mental health outcomes, with improved provision linked to better average mental health scores. However, this result was not significant and so results must be taken with caution.

## 4. Discussion

There has been a continued reported increase in mental health difficulties faced by young people, with 10.8% of 5–16-year-olds with a probable mental disorder in 2017 [1] rising to 16.0% in 2020 [3]. This can be linked to a range of factors such as increased school stress and exam pressure, peer relationships, social media, and poverty [34]. More recently, the COVID-19 pandemic and subsequent lockdowns has also impacted the mental health of young people in the UK [35]. Although the full effect of this is not yet known, a recent survey of young people showed 67% believed the pandemic would have a long-term negative effect for their mental health [36].

A whole school approach involves staff, children, and the community [37] and would be expected to be associated with increased mental health awareness and support across the school environment. This is suggested to be because of involvement of multiple groups (parents, teachers, pupils, the community), enabling positive culture change [16,21]. Relevant elements of the whole school approach include integrating mental health into the wider curriculum, training staff in mental health awareness, developing specific school policies related to mental health, implementing peer support systems, supporting parents, and utilising strong links with outside support agencies. Although 71% of schools included in the survey reported that they believed they had a whole school approach, none of the schools included all six of the criteria and only 43% achieved at least four. This suggests wide differences in the interpretation of what a whole school approach means. How a whole school approach is defined needs more clarification, as seen by the wide variability in school services. This links to literature previously discussed which also highlights a need for improved evaluation and reporting [26].

All schools in this study provided some level of universal provision, with 100% of schools reporting students being able to access members of the pastoral team for mental health support, although schools varied greatly in the size of their pastoral support teams. Of all 21 schools included, only one had a specific wellbeing or mental health policy. For most schools, there was either no specific policy or aspects were integrated into existing SEN policies. Many schools also embed mental health education within the curriculum, promote exercise, and/or engage with voluntary services and the local community [22,23]. Although services should be flexible and tailored to specific need, there are few statutory baseline requirements and mental health provision is not assessed by Ofsted.

Despite increase in need and the introduction of new services in schools, there remains a lack of clear reporting and evaluation. In preparing this paper, the research team contacted both the Department for Education and the Department for Health and Social Care regarding mental health services and interventions offered in UK secondary schools between 2004 and 2015. Both departments responded to say they do not collect or hold this information. There is a strong need to improve methodologies for recording and evaluating mental health support in schools to ensure the most helpful approaches are used.

When comparing the results regarding provision and pupil mental health outcomes, we found a non-significant result for the Spearman’s correlation between school-based mental health provision and self-reported mental health scores. It is unlikely that provision alone would have a large impact on mental health outcomes of pupils, given the wide range of factors which also have an impact. Future research should take this into consideration when examining the impact of different school-based services.

For example, there are additional factors that should be included in future research examining school-based mental health support. Reports highlight the links between pupils classed as disadvantaged (defined as eligible for free school meals or pupil premium funding) and negative outcomes in terms of inequalities, poorer mental health, and poorer school attainment [7]. Geographical areas may also play a role in terms of rural, urban, and coastal areas. There are differences between males and females in terms of mental health difficulties [27] and research has shown that some groups, such as those from ethnic minority populations, those who identify as LGBTQ+ or Looked After Children, may be at higher risk of developing mental health problems [38,39,40].

It was beyond the scope of this current survey to examine all of these factors in detail; however, future work should focus on this. There is a need for robust research to collect detailed information around geographical, socio-economic factors, and demographics and link these to both school provision and mental health. Further research needs to focus on what type of support works best for different pupils and how best to deliver it.

The results from this survey should be interpreted with caution, given it includes a relatively small sample and the same interventions are likely to be implemented differently in different schools. As with many studies using self-reported questionnaire outcome measures, there is the possibility of recall bias. Future research following on from this study should aim to address this. For example, it may be more appropriate to collect multi-informant SDQ outcomes. The current staff questionnaire may not have sufficient detail to gather the depth and range of provision available. There is a complex pattern of service provision across schools and improved definitions of what a whole school approach should look like and better recording is needed to enable more effective evaluation.

Despite this, the overall scores give an estimate of the range of available services and the potential impact of this on pupil mental health, as well as providing a baseline from which to give direction for future research. This study presents preliminary data into a current and timely area; pupil mental health and school-based support. With high numbers of young people experiencing mental health difficulties and in need of support, improvements to school-based provision could have a significant positive impact. This is a complex area, with future robust research urgently needed to identify how school-based support can be best implemented to support young people in need.

## 5. Conclusions

This study provides an overview of mental health services currently offered in schools and their potential impact on student mental health. It highlights the need for improved reporting and evaluation. Future services should take into account varying school needs and the wide range of factors that impact young people’s mental health. With increasing numbers of young people experiencing mental health difficulties, school-based provision could have a significant positive impact in supporting them. There is a strong need for further research in this area to identify which services are effective for which pupils and to improve evaluation of school-based provision.

## Figures and Tables

**Table 1:** Six common support areas for school provision.

Support Area Identified	Description	Scoring
Number of staff in pastoral team	How many staff members were included in the pastoral team	0 = none in the pastoral team 1 = 1–3 members 2 = 4–9 members 3 = >10
Training in schools	Including mental health first aid training for existing members of staff	0 = no trained members of staff 1 = 1–9 trained members of staff 2 = 10–49 trained members of staff 3 = >50 trained members of staff
External therapeutic support	An external professional visiting the school such as a counsellor, ‘wellbeing worker’, or mental health professional who is not consistently based on the school site	0 = no external support 1 = consultation work only 2 = group or 1:1 work 3 = group and 1:1 work with this external professional
Internal trained mental health staff	Includes professional mental health workers who are based within the school	Scored 0 or 1 according to whether this service was available or not
Voluntary agency	Includes any external charity or organisation that goes into the school to raise awareness or deliver work, for example MIND. This agency may visit a school to present during assemblies or to work specifically with individual pupils	Scored 0 or 1 according to whether this service was available or not
Peer mentor	This involves linking up pupils in schools, usually older pupils with younger pupils to provide extra support	Scored 0 or 1 according to whether a peer mentor scheme was available or not

**Table 2:** Demographics of respondents.

Characteristic	*n* (%)
**Gender**	
Male	2443 (39)
Female	2827 (45)
Prefer not to say/not completed	1058 (17)
**Year Group**	
8	2907 (46)
9	1711 (27)
11	1603 (25)
Not completed	107 (2)

**Table 3:** Pupil completed SDQ results.

SDQ Scale	Mean (SD)	‘Very High’	*n*
Total Difficulties	11.3 (5.9)	593 (9%)	6305
Emotion	3.4 (2.5)	866 (14%)	6315
Conduct	1.1 (1.8)	267 (4%)	6313
Hyperactivity	4.1 (2.5)	674 (11%)	6310
Peer problems	1.9 (1.7)	511 (8%)	6310
Prosocial	7.0 (2.0)	707 (11%)	6323
Impact	0.7 (1.5)	687 (11%)	6209

**Table 4:** Common support items.

Common Support Items	Percentage of Schools Offering Service	Details
Pastoral care team	100%	Reported numbers of staff in the pastoral team ranged 1–18
Training for school staff	95% had teachers trained in Mental Health First Aid	Numbers of trained staff ranged from 1 to ‘all staff members’ which included over 80 individuals
External therapeutic support	90% report some variation of external support	76% of schools accessed a ‘wellbeing worker’ as part of their external provision43% utilised links with an external organisation
Internal trained mental health member of staff	86%	Of these schools, this included either a school counsellor (11%), educational psychologist (50%), or both (39%)
Voluntary agency	57%	Includes charities focused on mental health and domestic abuse (e.g., MIND, Samaritans), local authority family support services, bereavement support, mentoring schemes, and mediation counselling. These services worked with specific pupils who were referred to them for support, and also delivered group sessions such as whole school assemblies
Peer mentoring	43%	Including peer mentoring, and linking up younger and older pupils for specific sessions
**Other**		
PSHE targeted at mental health	81%	Schools noted these are often delivered by staff with limited training
Mental health promotion across the curriculum through other lessons	48%	In physical education, ICT, science
Work with parents around mental health support	86%	Varying from signposting to external services through to meetings, workshops, and individual support, including direct referrals to professional services
Believe their school has a whole school approach	71%	-

## Data Availability

The data presented in this study may be available on request from the corresponding author. The data are not publicly available due to ethical reasons.
